# Relationships between *emm *and multilocus sequence types within a global collection of *Streptococcus pyogenes*

**DOI:** 10.1186/1471-2180-8-59

**Published:** 2008-04-11

**Authors:** Debra E Bessen, Karen F McGregor, Adrian M Whatmore

**Affiliations:** 1 Department of Microbiology and Immunology, New York Medical College, Valhalla, NY, 10595, USA; 2 Department of Infectious Disease Epidemiology, Imperial College London, UK; 3 Department of Bacterial Diseases, Veterinary Laboratories Agency, Addlestone, Surrey, UK; 4 Microbiology Research Group, Thames Valley University, London, UK

## Abstract

**Background:**

The M type-specific surface protein antigens encoded by the 5' end of *emm *genes are targets of protective host immunity and attractive vaccine candidates against infection by *Streptococcus pyogenes*, a global human pathogen. A history of genetic change in *emm *was evaluated for a worldwide collection of > 500 *S. pyogenes *isolates that were defined for genetic background by multilocus sequence typing of housekeeping genes.

**Results:**

Organisms were categorized by genotypes that roughly correspond to throat specialists, skin specialists, and generalists often recovered from infections at either tissue site. Recovery of distant clones sharing the same *emm *type was ~4-fold higher for skin specialists and generalists, as compared to throat specialists. Importantly, *emm *type was often a poor marker for clone. Recovery of clones that underwent recombinational replacement with a new *emm *type was most evident for the throat and skin specialists. The average ratio of nonsynonymous substitutions per nonsynonymous site (Ka) and synonymous substitutions per synonymous site (Ks) was 4.9, 1.5 and 1.3 for *emm *types of the throat specialist, skin specialist and generalist groups, respectively.

**Conclusion:**

Data indicate that the relationships between *emm *type and genetic background differ among the three host tissue-related groups, and that the selection pressures acting on *emm *appear to be strongest for the throat specialists. Since positive selection is likely due in part to a protective host immune response, the findings may have important implications for vaccine design and vaccination strategies.

## Background

A molecular arms race between pathogen and host often emerges when an immune response favors the selection of microorganisms displaying altered antigens on their surface. The mechanisms by which bacterial pathogens undergo immune escape include point mutation and replacement of antigen genes by homologous recombination following horizontal transfer of DNA between organisms of different strains. Genetic change provides the raw material upon which natural selection acts, and a host immune response is one of the strongest selection pressures a microbial pathogen will encounter.

The fibrillar M protein molecule present on the surface of *Streptococcus pyogenes*, a bacterial pathogen afflicting humans throughout the world, is often the target of a protective immune response mounted during infection [1-3]. M protein is an essential virulence factor and provides the basis for serotype [4]. A more recent typing scheme based on the nucleotide (nt) sequence at the 5' end of the *emm *gene, encoding the distal fibril tip, closely parallels serologic findings and has led to the identification of ~160 *emm *types [5]. Importantly, strong protective immunity to *S. pyogenes *infection is often M type-specific.

*S. pyogenes *strains can be divided into 3 major groups that roughly correspond to preferred tissue site for infection. Historically, it has been recognized that certain M types are strongly associated with cases of pharyngitis, whereas other M types are more often recovered from superficial skin infections (i.e., impetigo) [6-8]. Genotypic markers – known as *emm *patterns – are based on the phylogeny of the 3' end of *emm *genes, encoding the semi-conserved cell wall-spanning domain of M protein [9,10]. Of biological relevance is the finding that *emm *pattern genotypes display strong associations with strains causing superficial infections at the throat or skin, whereby *emm *pattern A-C strains tend to cause pharyngitis (referred to as throat specialists), pattern D strains tend to cause impetigo (skin specialists), and pattern E strains as a group are often found in association with infections at both tissues (generalists) [11,12]. Data from 11 population-based surveillance studies on streptococcal pharyngitis and/or impetigo, spanning all 6 major continents, provide strong support for these biological groupings (Table 1), even though tissue associations are not strict and occasionally deviate, particularly in communities having high rates of both pharyngitis and impetigo [11,13-20]. Also, organisms found in association with throat carriage (versus infection) may not correlate as well with the *emm *pattern groupings [11,17].

**Table 1:** Population-based surveillance of *S. pyogenes*.*

**Location of surveillance**	**No. of *S. pyogenes *isolates**	**% of pharyngitis (or tonsillitis) isolates that are *emm *pattern:**			**% of impetigo isolates that are *emm *pattern:**		
		**A-C**	**D**	**E**	**A-C**	**D**	**E**

Australia, tropical	125	NR	NR	NR	13	46	41
Rome (Italy)	114	50	1	48	n/a	n/a	n/a
Germany	216	51	0	49	n/a	n/a	n/a
Spain	520	32	1	68	n/a	n/a	n/a
Mexico	282	54	1	44	n/a	n/a	n/a
USA	> 1,900	53	1	47	n/a	n/a	n/a
Ethiopia	104	26	28	35	0	32	47
Nepal	53	NR	NR	NR	19	30	51
Brazil	87	20	20	61	3	55	42
Brussels (Belgium)	163	55	0	45	NR	NR	NR
Australia, tropical	129	NR	NR	NR	13	53	35

* *emm *pattern is inferred based on *emm*-type (McGregor et al., 2004; reference 23) NR, < 10 isolates recovered; n/a, not applicable.

Extensive analysis of multilocus sequence typing (MLST) data for *S. pyogenes *strains assigned to the *emm *pattern-defined groups indicates that there is a history of ample flow of housekeeping genes between the 3 groups [21,22]. Furthermore, there is a lack of housekeeping gene sequence clustering among isolates derived from patients known to have throat versus skin infection. The *emm *pattern-defined groups do not appear to represent deep ancestral lineages of *S. pyogenes*, based either on concatenated housekeeping gene trees or individual housekeeping gene tree topologies. Yet, for > 98% of *emm *types, all isolates examined that share an *emm *type are assigned to the same *emm *pattern group [23], suggesting that a given *emm *type is largely restricted to a single *emm *pattern group; this finding was recently validated in another study [20]. Experiments in which *emm *genes are swapped between strains of different *emm *pattern groups show that M protein function depends on interactions with other cell factors [24,25]. Several *emm *pattern-linked traits encoded by physically distant loci have been identified and they may work in concert to play a critical role in adaptation of the organism to different ecological niches [26-29].

*S. pyogenes *is responsible for a large global burden of disease, and development of a preventative vaccine is a high priority [3,30,31]. The strong protective immunity elicited by M type-specific epitopes has led to efforts to develop an M type-based vaccine. In this report, genetic changes in *emm *type – due to mutation and/or recombination – are evaluated for strains defined for tissue site preference of infection.

## Results

### Characteristics of the strain sample set

Infection type is defined by a clear set of clinical criteria in each of the population-based surveillance studies listed in Table 1, upon which the strength of the association between infection type (pharyngitis, impetigo) and *emm *pattern genotype rests. However, in order to rigorously address the relationship between *emm *and genetic background on a global scale, a genetically diverse set of organisms spanning a wide time frame and geographic space is required instead. A genetically diverse set of *S. pyogenes *strains, isolated from > 25 countries throughout the world, was assembled for analysis (see Additional file 1).

Nucleotide (nt) sequence data was obtained for 7 housekeeping loci, providing ST assignments, and for the type-specific region of *emm *positioned at the 5' end of the locus, providing *emm *type and *emm *allele assignments. MLST and *emm *typing data was previously reported for 493 of the isolates under study [23,32,33]; an additional 89 isolates were included based on their large geographic distance relative to isolates sharing that *emm *type; *emm *alleles were determined for the majority of isolates (see Additional file 1).

The complete data set contains 582 isolates represented by 259 sequence types (STs) and 156 *emm *types (Table 2). ST is used as a marker to distinguish among isolates with different genetic backgrounds. Approximately 97% of the known *emm *types of *S. pyogenes *[34] are included in the sample set. Nearly all of the isolates (577 of 582) can be assigned to one of the 3 *emm *pattern groups [23], a genotype that corresponds well to throat specialists (pattern A-C; N = 156), skin specialists (pattern D; N = 181) and generalists (pattern E; N = 240).

**Table 2:** Summary of *S. pyogenes *sample (sub)sets analyzed in this study.

			Distribution according to emm pattern:			
**Sample (sub)set**	**Characteristic**	**Total**	**A-C**	**D**	**E**	Undefined or other

Complete	Number of isolates	582	156	181	240	5
Complete	Number of STs represented	259	42	91	123	5
Complete	Number of emm types represented	156	29	62	61	4
Complete	Number of unique combinations of emm type and ST	280	47	104	124	5
Complete	Simpson's diversity index *	0.993	0.950	0.985	0.990	n.d.
Complete	Simpson's diversity index, 95% confidence intervals	0.992–0.995	0.938–0.963	0.979–0.991	0.987–0.992	n.d.
emm nt substitution	Number of isolates	520	137	155	220	8
emm nt substitution	Number of emm types represented	105	18	40	44	3
emm nt substitution	Number of emm alleles represented	188	54	57	71	6
emm HGT	Number of isolates	531	143	156	224	8
emm HGT	Number of emm types represented	105	18	40	44	3
emm HGT	Number of STs represented	219	34	75	108	2

* Based on unique combinations of *emm *type and ST. Abbreviations: n.d., not determined; HGT, horizontal gene transfer; nt, nucleotide; ST, sequence type

Each *emm *pattern-defined group is highly diverse, as evidenced by Simpson's diversity index (*D*) values of 0.950, 0.985 and 0.990 for *emm *pattern A-C, D and E isolates, respectively. For the *D *value calculation, *S. pyogenes *clones are defined by their combination of *emm *type and ST. A *D *value equal to one signifies that the genotyping method distinguishes between all isolates, whereas a *D *value equal to zero means that all isolates are the same clone.

In summary, the strain sample set is characterized as being both comprehensive, including representatives of most known genotypes, and highly diverse, containing relatively few isolates that represent identical clones.

### Diversifying selection in *emm *alleles

Because the M type-specific region is a target of a protective immune response by the human host [1,2], nt substitutions at nonsynonymous sites and insertions or deletions (indels) have the potential to modify the antigenic structure of the surface protein and render an immune response ineffective.

Alignment of nt sequences assigned to the same *emm *type were generated by Clustal W. Fifty-one of the 582 isolates corresponded to *emm *types that are unique to a single isolate and were not aligned (see Additional file 1). Two or more isolates were sampled for 105 *emm *types (see Additional file 2). The 105 separate alignments include *emm *sequences from 520 isolates (11 of the 531 sequences were not assessed), represented by 189 distinct *emm *alleles. For each of the 105 Clustal W alignments of *emm *type, whereby each alignment contains 150 nt sites and at least 2 *emm *sequences, Ka and Ks values were calculated. Nonsynonymous substitutions (measured by Ka) result in an amino acid change, whereas synonymous substitutions (measured by Ks) are silent. Since 58 of the 105 *emm *type alignments were devoid of nt polymorphisms, the average mean of the Ka and Ks values for all 105 alignments was determined. The ratio of the average mean Ka value to the average mean Ks value was 1.96 (Table 3), indicative of positive diversifying selection acting on the type-specific region of *emm *genes.

**Table 3:** Synonymous and nonsynonymous nucleotide substitutions within the *emm *type region (150 nt), based on Clustal W alignments corresponding to each *emm *type

emm pattern	No. of emm types (%)	No. of isolates analyzed	Average no. of nonsynonymous substitutions per nonsynonymous site (Ka) #	Average no. of synonymous substitutions per synonymous site (Ks)	Average ratio of Ka to Ks
A-C	18 (17)	137	0.02121	0.00431	4.92
D	40 (38)	155	0.00748	0.00488	1.53
E	44 (42) *	220	0.00732	0.00581	1.26
other^	3	8	0.02633	0.00721	3.65
All	105 (100)	520 $	0.01037	0.00526	1.96

# Represents average of values from alignments corresponding to each *emm *type

$ Sequence alignment of (partial) *emm *genes was performed for 520 of the 531 isolates.

* *emmst206 *is included with emm96 analysis since the large indel (54 nt) is at the extreme 5' end.

^Includes two *emm *types associated with multiple *emm *patterns (*emm54, emmst854*; patterns A-C and D) and *emm31 *(pattern uncertain).

The impact of diversifying selection was next examined for *emm *types in accordance with *emm *pattern group. The average mean of the Ka and Ks values was calculated and yielded a Ka to Ks ratio of 4.92 for the pattern A-C subset of *emm *types, but only 1.53 and 1.26 for the patterns D and E groups, respectively (Table 3). Pair wise comparisons between raw Ka and Ks values were significantly different for the pattern A-C *emm *types (*t *< 0.01, paired *t*-test, 2 tailed), but not for the pattern D or E *emm *types (see Additional file 2). Also, the raw Ka values were significantly different for pattern A-C *emm *types versus either pattern D or E *emm *types (*t *< 0.05, unpaired *t-*test, 2-tailed); no significant differences were found for the Ks values.

The average mean Ka to Ks ratio for pattern A-C *emm *types exceeded the values observed for pattern D and E *emm *types by 3- to 4-fold. The data provide evidence that diversifying selection is strongest for pattern A-C *emm *types. Because the *emm *types associated with *S. pyogenes *are largely restricted to this bacterial species [35,36], the observed genetic changes most likely originated as mutations within the *S. pyogenes *population, rather than having arisen by lateral transfer from another species.

Small indels within the *emm *type-specific region can lead to alterations in the phenotypic surface expression of M protein. Only 2 isolates belonging to the complete data set – both of which are pattern A-C and not included among the 520 isolates comprising the *emm *gene sequence alignments – have indels within the *emm *type region that lead to frame shift mutations and premature termination of the translated M protein products (see Additional file 2). Conceivably, loss of M protein via a frame shift may be a strategy for immune escape that is largely restricted to pattern A-C strains however, the number of events is too small to draw conclusions.

In-frame indels are observed in 11 (10%) of the 105 *emm *type alignments. Four and 7 pattern D and E *emm *type alignments, respectively, each contain 1 allele having an indel (see Additional file 2). Indels most likely arise via slipped strand mispairing during DNA replication or homologous recombination resulting in an unequal crossover. Thus, epitope loss or gain mediated via small indels may constitute a strategy used by a small proportion of pattern D and E strains to alter antigenic structure and evade a specific immune response.

### Recombination involving *emm *type: STs associated with multiple *emm *types

An important strategy for escape from a protective host immune response directed towards M protein is the recombinational replacement of an *emm *type following a horizontal gene transfer (HGT) event, whereby the donor and recipient strains differ in *emm *type. In general terms, interstrain gene exchange is favored by close physical proximity between the donor and recipient cells, which may occur during a co-infection taking place at either the throat or skin [6,37].

Of the 259 STs identified in the set of 582 isolates, 14 STs were recovered in association with > 1 *emm *type; they are referred to as *emm*-variable STs. Although the 14 *emm*-variable STs account for only a small percentage of the total STs (5.4%), > 20% of the *emm *types (N = 35) were found in association with these few STs (Table 4). The *emm *pattern D subset had the greatest number of *emm*-variable STs (N = 9), which collectively, were recovered in association with 23 different *emm *types. Only 6.6% of the *emm *types assigned to pattern E were present among *emm*-variable STs. The number of recombinational replacements of *emm *type per ST was at least 7- to 9-fold higher for the patterns A-C and D subsets (0.119 and 0.154, respectively) as compared to pattern E strains (0.016) (Table 4). The data are consistent with the idea that substitution with a completely new *emm *type may be an important adaptive strategy for the specialist strains, and less critical for the generalists.

**Table 4:** Recombinational replacement of *emm *with a new *emm *type.

emm pattern	No, of isolates	No. of STs	No. of emm type-variable STs (%)	No. of emm types *	No. of emm types associated with emm type variable STs (%)	No. of recombinational events	No. of recombinational events per locus per ST
A-C	156	42	3 (7.1)	29	8 (27.6)	5	0.119
D	181	91	9 (9.9)	62	23 (37.1)	14	0.154
E	240	123	2 (1.6)	61	4 (6.6)	2	0.016
All	577	256	14	152	35	21	0.096 average

* One ST scored as pattern D (ST3) and 1 ST scored as pattern E (ST150) contain isolates with rearranged *emm *region yielding a new *emm *type, but could not be assigned a pattern; they are listed here with the pattern D and E groups, respectively. Other *emm *pattern-undefined isolates are not included in chart.

### Recombination involving *emm *type: *emm *types on distant STs

The same *emm *type present on STs differing at ≥ 5 housekeeping alleles can arise from HGT of *emm *to a distant ST or alternatively, by genetic changes in most housekeeping loci in the absence of a corresponding shift in *emm *type assignment. Recovery of *emm *types on STs of intermediate genetic distances, or the lack thereof, can help to distinguish between horizontal movement of an *emm *type to a distant ST versus genetic diversification at most housekeeping loci.

**Figure 1:**
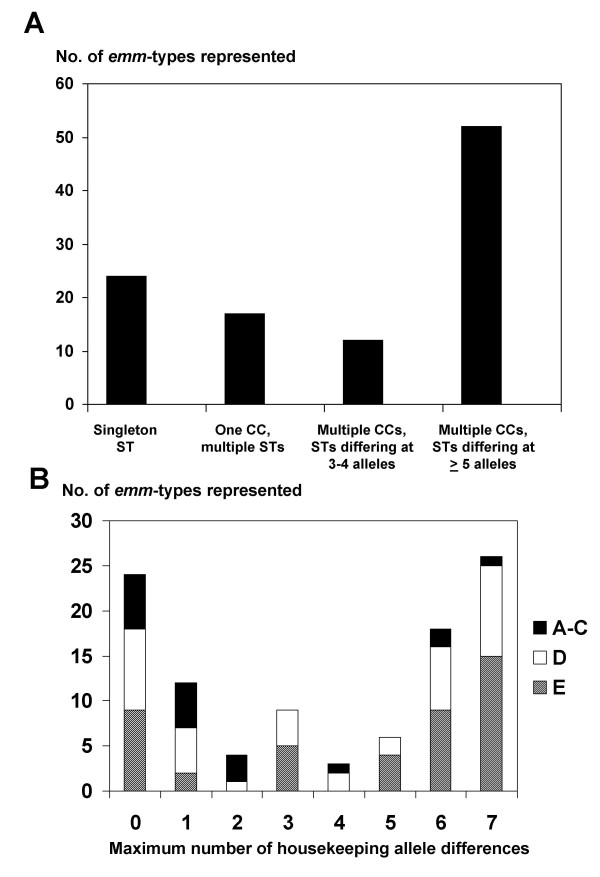
**Differences in the number of housekeeping alleles between isolates sharing an *emm *type**. The y-axis shows the numbers of *emm *types represented by are each category, as defined by the x-axis. (A), The minimum number of differences in housekeeping alleles between isolates sharing an *emm *type are: zero (singleton STs), one or two (1 CC with multiple STs), three or four (multiple CCs with STs of intermediate distance), and five (multiple CCs whereby all STs are distant; represents HGT). (B), Distribution of the maximum number of differences in housekeeping alleles between isolates sharing an *emm *type. Clonal complex (CC) is defined by STs sharing at least 5 of 7 housekeeping alleles

The 105 *emm *types having ≥ 2 isolates represented, and together comprising a sample set of 531 isolates, were examined for the distribution of *emm *type among different STs (Figure 1A). Twenty-four *emm *types were exclusively associated with a single ST. Another 17 *emm *types were associated with multiple STs that were assigned to the same clonal complex (CC), defined as sharing ≥ 5 of the 7 housekeeping alleles. Thus, for these 41 *emm *types, there is no evidence for HGT of *emm *type to a distant genetic background.

In contrast to the above findings, 52 *emm *types were found in association with distant STs and ≥ 2 CCs (Figure 1A), whereby ≥ 5 housekeeping allele differences are evident for all possible pair wise comparisons of the STs assigned to different CCs. Distant STs are defined as those differing at ≥ 5 housekeeping alleles. Since intermediate MLST genotypes could not be identified among organisms sharing these 52 *emm *types, it is reasonably argued that they likely arose via horizontal transfer of an *emm *type to a distant ST in a single genetic step, rather than by diversification of an ST at ≥ 5 housekeeping loci involving ≥ 5 independent genetic steps.

Among the 52 *emm *types associated with distant STs, 63 HGT events to distant STs could be distinguished (Table 5). About half (51%) of the HGT events involved identical *emm *alleles and therefore, may have occurred within the relatively recent evolutionary past.

**Table 5:** *emm *types associated with distant STs.

emm pattern	No. of emm types examined *	No. of emm types involved in HGT	No. of HGT events involving emm type	Average no. of HGT events per emm type	Average no. of isolates sampled per emm type	Average no. of countries sampled per emm type	No. of HGT events involving the same emm allele	No. of emm types restricted to 1 ST or CC
A-C	18	3	3	0.17	7.94	2.78	2	14
D	40	19^	24^	0.60	3.90	2.18	15	15
E	44	27	33	0.75	5.09	2.28	15	12
other	3	3	3	1.00	2.67	1.67	0	0
Total	105	52	63	n/a	n/a	n/a	32	41

* *emm *types examined are those whereby 2 or more isolates are present in the sample set (i.e., sample set of 531 isolates, excluding the 51 singletons).

^ One *emm *type (*emm93*) may not be involved in HGT based on identification of an intermediate ST in another study [49]

Abbreviations: n/a, applicable; CC, clonal complex; HGT, horizontal gene transfer; ST, sequence type

Only 12 *emm *types are associated with STs that were assigned to 2 CCs that differ in as few as 3 or 4 housekeeping alleles (i.e., STs of an intermediate genetic distance) (Figure 1A). Therefore, the majority of *emm *types were either restricted to a single CC (39%) or associated only with distant STs (50%), with relatively few *emm *types falling into the intermediate category (11%). This finding is further underscored by the distribution of the maximum number of differences in the 7 housekeeping alleles between STs sharing an *emm *type (Figure 1B). Thirty-six (34%) of the *emm *types are associated with STs having 0 or 1 housekeeping gene difference, whereas 47 (45%) *emm *types are associated with STs having 6 or 7 housekeeping gene differences. Only 21% of the *emm *types lie in between these two extremes, with 2, 3, 4 or 5 housekeeping gene differences.

The horizontal transfer of an *emm *type to a distant ST was compared for the 3 *emm *pattern-defined groups of isolates (Table 5). Only 3 of the 18 pattern A-C *emm *types (17%) were recovered in association with distant STs. In contrast, 48 and 61% of the pattern D and E *emm *types, respectively, displayed evidence for HGT. Eight *emm *types – 4 each from the pattern D and E groups – were found in association with ≥ 3 distant STs of distinct CCs, indicative of multiple HGT events involving these *emm *types. The average mean number of HGT events per *emm *type is calculated as 0.17, 0.60 and 0.75 for the pattern A-C, D and E groups, respectively.

Using a 2 × 2 test for independence (Fisher's exact, 2-tailed) with Table 5 data, the difference between the *emm *pattern A-C and D strain groups in terms of the number of *emm *types having undergone HGT, versus restriction to a single ST or CC, is significant (*p *= 0.015). The difference is highly significant between the *emm *pattern A-C and E strain groups (*p *< 0.005), but not significant when pattern D and E strains are compared.

The strikingly higher number of HGT events uncovered for pattern D and E *emm *types is not likely a consequence of sampling bias (Table 5). If there was sampling bias, it is expected that more extensive sampling of a given *emm *type would result in an increase in the number of HGT events detected simply by chance. Yet, the mean average number of isolates sampled per *emm *type was highest for pattern A-C strains, the group which displayed the fewest HGT events. The average number of countries sampled per *emm *type was slightly higher for the pattern A-C group as compared to the other subsets (Table 5) and therefore, geographic distance does not appear to explain the higher levels of HGT observed for the pattern D and E groups. For *emm *types with no HGT detected, irrespective of *emm *pattern group, an average of 2.25 (s.d., 1.24) countries were sampled per *emm *type, whereas for *emm *types displaying ≥ 1 HGT event, a similar average of 2.38 (s.d., 0.796) countries were sampled per *emm *type (data not shown).

The findings on HGT of *emm *indicate that recovery of the same *emm *type on distant STs is most probable for isolates belonging to the skin specialist (pattern D) and generalist (pattern E) groups. Importantly, the data also show that *emm *type can be a poor marker for clone (i.e., ST) or clonal complex (CC). The higher prevalence of HGT of *emm *type among the pattern D and E groups may be a consequence of higher intrinsic recombination rates, different selection pressures and/or a combination of both genetic change and selection effects.

### Estimate of relative levels of recombination based on housekeeping genes

Recovery of STs associated with multiple *emm *types was highest for throat specialists (Table 4), whereas recovery of *emm *types associated with multiple STs was highest for generalists (Table 5). Estimates of the relative levels of recombination among housekeeping genes for the *emm *pattern-defined groups might help to discern between the contribution of genetic change and the effects of selection. The Ka to Ks ratios for each of the 7 housekeeping genes is less than one (range, 0.033 to 0.493; data not shown), and lower than the values obtained for *emm *type sequences (Table 3).

eBURST is a clustering algorithm that has been widely used to assess the mechanisms of genetic change within a bacterial population, based on MLST of housekeeping genes [38]. An estimate of the number of recombination versus mutations events can be made based on the nature of the nt differences between the mismatched allele of a single locus variant (SLV) pair. In a previous report on *S. pyogenes*, ≥ 28 of the 48 SLVs were attributed to recombination in accordance with a count-based method that was conservative for scoring recombination [23]. In this larger strain sample set of 582 isolates, the same approach used in the prior study was applied; 56 SLVs were detected by eBURST, wherein ≥ 33 genetic changes were estimated to have arisen following recombination (Table 6).

**Table 6:** Estimate for the minimum number of recombinational events involving housekeeping genes.

emm pattern	No. of STs	No. of recombinational events	No. of loci	No. of recombinational events per locus per ST
A-C	42	2	7	0.007
D	91	16	7	0.025
E	123	15	7	0.017

The minimum number of recombination events per housekeeping locus per ST was calculated as 0.007, 0.025 and 0.017 for *emm *pattern groups A-C, D and E, respectively (Table 6). These findings are in agreement with the general trend from previous estimates of recombination based on the congruency of housekeeping gene tree topologies [22], whereby *emm *pattern A-C strains showed the highest level of congruence and thereby, the lowest relative level of recombination, and *emm *pattern D strains displayed the lowest level of congruence and highest recombination [22]. Thus, based on the conservative estimates derived from SLV pairs, the relative rate of intrinsic recombination appears to be lower for the *emm *pattern-defined throat specialist group.

### Recombination involving *emm *type: Recovery of donors and recipients

*emm*-variable STs represent new clones and their progenitors (i.e., the recipient), whereas distant STs sharing an *emm *type represent new clones and their donors. The recovery of all 3 genotypes involved in the HGT event – donor, recipient, and new clone – from the extant *S. pyogenes *population was evaluated, based on the findings presented in Tables 3 and 4.

For the 8 pattern A-C *emm *types associated with 3 *emm*-variable STs, it is expected that 5 of the 8 *emm *types will have originated from donor strains; however, only 1 donor *emm *type (*emm14*) was recovered on a distant ST (20%). In contrast, of the 4 pattern E *emm *types associated with 2 *emm*-variable STs, potential donor strains were recovered for both HGT events (100%). Among pattern D strains, 23 *emm *types were recovered in association with 9 *emm*-variable STs (Table 4) and therefore, 14 donor *emm *types are to be expected; 9 *emm *types (64% of the total possible) were found on distant STs representing putative donors (see Additional file 1).

The number of recipient STs recovered from the natural population can also be evaluated for distant ST pairs that share the same *emm *type. Only 3 distant ST pairs sharing the same pattern A-C *emm *type were found among the collection of 582 strains, and a potential recipient ST was recovered for one of them (33%). A similar proportion (38%) of recipient STs were recovered for the 24 HGT events involving pattern D strains. In contrast, very few (2 of 33; 6%) recipient STs matching the pair of the pattern E *emm *donor and new clone were found.

The findings show that the proportion of STs associated with multiple *emm *types was highest for throat specialists, whereas the proportion of *emm *types associated with distant STs was highest for generalists. Although some of the numbers are quite small, the data reveal a trend whereby relatively fewer genotypes corresponding to pattern A-C donor and pattern E recipient strains were recovered by sampling than were expected.

## Discussion

*S. pyogenes *strains that are grouped according *emm *pattern genotype share a predilection for causing infection at particular tissue sites (Table 1). Analysis of *emm *pattern-defined strains for relationships between *emm *type and housekeeping genes reveals that the throat infection specialist group is distinct from the skin infection specialists and generalists in several key characteristics. This trend provides support for the idea that strains which tend to cause pharyngitis display different evolutionary dynamics.

Despite the possibility of underestimating positive selection using Ka to Ks ratios, the average ratios of Ka to Ks > 1 observed for *emm *type sequences of each *emm *pattern group provide evidence for positive diversifying selection. A likely source of the selection pressure is host immunity, whereby amino acid changes within the M type-specific region lead to alterations in antigenic structure that allow mutants to escape the protective immune response in at least some hosts. An important mechanism underlying protective immunity against *S. pyogenes *infection involves M type-specific antibodies that mediate opsonization and thereby, overcome the antiphagocytic property of M protein [1].

There is direct experimental evidence in support of amino acid changes in the M type-specific region that allow for immune escape [39-41]; this mechanism may even explain the recent emergence of an important M3 type clone. However, other findings that measure opsonophagocytosis with hyperimmune rabbit sera raised to the polypeptide product of one *emm *allele show high levels of bactericidal activity for strains of numerous *emm *alleles of the same *emm *type, arguing against the likelihood of immune escape mutants emerging in a vaccinated population [42]. Combined with the population genetics findings of this report, there is support for the notion that in a naturally infected human host population, the immune selection pressures on *emm *type may be somewhat lower in intensity and/or the immune response more variable in specificity, as compared to what can potentially be achieved in a vaccinated population.

The average ratio of Ka to Ks is ~3- to 4-fold higher for the *emm *pattern A-C group of *emm *types, suggesting that the throat specialists may be subject to stronger positive selection pressures. Several experimental findings may help to explain the lower Ka to Ks ratios observed for the skin specialists and generalists. A serological typing scheme based on the serum opacity reaction was developed as an alternative to M serotyping in order to circumvent difficulties encountered in determining the M type for many *emm *pattern E strains [10,43,44]; perhaps *emm *pattern E strains are more difficult to M type because of weaker immunogenicity and/or higher cross-reactivity. Hyperimmune rabbit antiserum raised to M type-specific antigens show that more pattern A-C *emm *types consistently elicit antiserum having a strong bactericidal effect, as compared to pattern E *emm *types [45]. The M type-specific regions of most *S. pyogenes *strains bind the complement regulator C4b-binding protein (C4BP). C4BP binding is achieved in the absence of a shared amino acid sequence motif and even though substitutions can introduce antigenic change without altering C4BP binding activity [46], it is conceivable that there are some functional constraints on sequence variation. Of probable relevance is the finding that most isolates lacking C4BP binding activity have *emm *types characteristic of pattern A-C strains (egs., M types 1, 3, 5, 6, 12, 19, 24, 26, 30, 39) [23,46], suggestive of higher levels of purifying (negative) selection on pattern D and E *emm *types in order to maintain C4BP binding activity.

For *emm *variable STs, whereby ST is defined by 7 housekeeping alleles, *emm *type can be regarded as an 8^th ^locus, wherein the related organisms are SLVs that arose via recombinational replacement of *emm*. Throat specialists appear to have the lowest level of recombination involving housekeeping genes, yet they display a relatively high number of recombinational replacements of *emm *type per ST. The observed imbalance in the relative levels of recombination affecting housekeeping genes versus *emm *genes supports the likely conclusion that pattern A-C *emm *types are subject to stronger positive selection. Thus, for throat specialists, immune escape mutants – arising by either mutation or recombination – appear to have a strong selective advantage, as compared to skin specialists and generalists.

The disproportionately low recovery rate for pattern A-C donor and pattern E recipient genotypes might be explained by several factors, which include competition between the new clone and the donor or recipient genotype as mediated through host or herd immunity [47]. For eg., if a new pattern A-C clone has a higher transmission rate than the donor genotype with which it shares an *emm *type, it may outcompete the donor by reducing the number of susceptible (i.e., nonimmune) hosts available to it. Likewise, if non-*emm *genes are critical for protective immunity against pattern E strains, the new clone may outcompete its progenitor if it has a higher transmission rate. Conceivably, immune-mediated competition may reduce the transmission success of the less fit organism to the point that it becomes rare and much more difficult to recover through sampling.

A previous study showed that many of the associations between *emm *type and ST differed for isolates collected from a remote Australian community, when compared to strains recovered from the United States and Europe [32]. The present study includes representatives of most known *emm *types, and demonstrates that *emm *type is a reasonably good marker for ST or CC among the throat specialist strains, but a rather poor marker for clone among skin specialists and generalists. This finding underscores the importance of multilocus typing methods for defining *S. pyogenes *strains [33].

The strong positive selection pressures acting on *emm *types of throat specialists are consistent with their role as targets of strongly protective immunity. Thus, an M type-based vaccine directed against pattern A-C strains is expected to have high efficacy, provided that the avenues for immune escape are blocked. This might be achieved by including numerous *emm *types in the vaccine formulation to help prevent the spread of new clones arising by recombinational replacement of *emm *type, and by eliciting a polyspecific immune response that protects against the spectrum of *emm *allelic variants that arise by mutation. Since only a small fraction of STs are associated with multiple *emm *types, targeting non-*emm *gene products of those STs, whereby the non-*emm *products elicit (partial) protection, could provide a sound complementary strategy.

The relatively weaker positive selection observed for *emm *types of pattern D and E strains is consistent with the possibility that they may be less ideal candidates for an M type-based vaccine. The pattern E generalists comprise about half of all known *emm *types [23], and account for ~40 to 50% of isolates collected in population-based surveys in many parts of the world (Table 1). An efficacious vaccine targeting this highly prevalent group of *S. pyogenes *organisms may require additional antigens in its formulation.

## Conclusion

This study provides a comprehensive population analysis of strains representing nearly all *emm *types; for the majority of *emm *types, multiple isolates recovered from distant geographic locations were studied. The relationships between *emm *type and genetic background differ among the 3 groups of *emm *pattern-defined genotypes which roughly correspond to host tissue site preferences for infection. Furthermore, the selection pressures acting on *emm *appear to be strongest for the throat specialists. Since a protective host immune response is probably a key factor driving positive selection, the findings provide important new insights that may aid in vaccine design and vaccination strategies.

## Methods

### Bacterial strains

Nearly all (493 of 495) isolates of *S. pyogenes *that were previously described in [23] are also included in this study. In an effort to expand the number of isolates sharing an *emm *type with isolates of the previous data set, and also having been recovered from a distant geographic location, 89 bacterial isolates representing 65 different *emm *types were added to the analysis, comprising the complete sample set of 582 isolates (see Additional file 1).

### emm based genotypes

*emm *type and *emm *allele were established by nucleotide (nt) sequence determination of PCR amplicons [5]. *emm *type is a character state whereby a unique *emm *type is defined as sharing < 92% sequence identity over the first 90 bases encoding the deduced processed M protein of the type reference strain, allowing for small indels [34]; *emm *type strongly correlates with M protein serotype, as previously established by immunoreactivity with typing sera [5,48]. *emm *allele assignments are based on the first 150 nt corresponding to the 5' end region encoding the mature M protein molecule [34]; each allele has a unique sequence, and is equivalent to "*emm *subtype." *emm *pattern was ascertained by PCR-based mapping, or was inferred based on *emm *type, as described [23].

### MLST

Internal fragments of 7 housekeeping genes (*gki, gtr, murI, mutS, recP, xpt, yqiL*) were amplified by PCR and the nt sequence determined using primers and conditions described previously [33]. For each locus, distinct allele numbers were assigned to each unique sequence, generating a seven integer allelic profile for each isolate; isolates with identical allelic profiles were assigned to the same ST. A complete database of alleles, allele sequences and STs is maintained on the internet [49]. A total of 38 new STs are reported.

### Computations

Simpson's diversity index (*D*) was calculated as described [50]. The 150 nt *emm *type-specific sequence derived from > 2 isolates assigned to the same *emm *type were aligned using the Clustal W algorithm, implemented in Megalign (DNASTAR, Lasergene, Inc.). The number of nonsynonymous substitutions per nonsynonymous site (Ka) and the number of synonymous substitutions per synonymous site (Ks) were calculated for each Clustal W alignment, using DnaSP version 4.10 [51]. The eBURST clustering algorithm [38] for analyzing relationships between STs was applied using software available at . Single locus variants (SLVs) were identified with a user-defined setting of 6 of 7 shared housekeeping alleles. The method for estimating recombination events was previously described [23].

## Authors' contributions

KM and AW generated and analyzed sequence data. DB conceived the study, participated in its design and coordination, generated and analyzed some sequence data, performed computational analysis, and wrote draft of the manuscript. All authors read and approved the final manuscript.

## Supplementary Material

Additional file 1 Characteristics of all 582 S. pyogenes isolates under study. The data provided represent numerous key characteristics of the strains under study. Click here for file

Additional file 2 Analysis of nt substitutions among alleles assigned to the same emm type. The data provided represent the raw data used to calculate values in Table 3. Click here for file
